# Cancer prevention in females with and without obesity: Does perceived and internalised weight bias determine cancer prevention behaviour?

**DOI:** 10.1186/s12905-022-02085-2

**Published:** 2022-12-09

**Authors:** Marie Bernard, Magrit Löbner, Florian Lordick, Anja Mehnert-Theuerkauf, Steffi G. Riedel-Heller, Claudia Luck-Sikorski

**Affiliations:** 1 Medical Faculty, Institute of Medical Sociology, Martin Luther University Halle-Wittenberg, Magdeburger Straße 8, 06112 Halle (Salle), Germany; 2 Integrated Research and Treatment Centre AdiposityDiseases (IFB), University of Leipzig, Leipzig, Germany; 3 SRH University of Applied Health Sciences, Gera, Germany; 4 Medical Faculty, Institute of Social Medicine, Occupational Health and Public Health, University of Leipzig, Leipzig, Germany; 5 Leipzig University Cancer Center, University of Leipzig Medical Center, Leipzig, Germany; 6 Department of Medical Psychology and Medical Sociology, University Hospital of Leipzig, Leipzig, Germany; 7 Helmholtz Zentrum München, German Research Center for Environmental Health, Helmholtz Institute for Metabolic, Obesity and Vascular Research (HI-MAG), Leipzig, Germany

**Keywords:** Cancer prevention, Obesity, Weight bias internalisation, Stigma

## Abstract

**Background:**

Women with obesity are not only at higher risk of developing cancer such as gynaecological malignancies but are also less likely to attend cancer prevention screenings (CPS). In this study, we aimed to obtain a better database for Germany and to investigate whether women with obesity are less likely to undergo CPS compared to women without obesity. Moreover, we aimed to identify factors that determine CPS behaviour.

**Methods:**

A quantitative cross-sectional telephone survey was conducted that assessed data of 1003 females in the general public with obesity (BMI ≥ 30 kg/m^2^; n = 500) and without obesity (BMI < 30 kg/m^2^; n = 503). We assessed participants’ utilisation of cervical, breast, and colorectal CPS. Group differences were investigated by using Chi-Square tests, whereas influencing factors that might determine CPS behaviour were examined by multivariate logistic regression analyses. Therefore, logistic regression models for (a) the full sample and (b) the obese sample were conducted. Explanatory factors (i.e., cancer awareness, the internalisation of weight bias (WBIS) and perceived weight-based discrimination) were included. Confounding factors such as sociodemographic variables were included in the multivariate analysis.

**Results:**

Women with obesity were less likely to undergo Pap smear (χ^2^(1) = 13.90, *p* < 0.001) and clinical breast examination (χ^2^(4) = 14.41, *p* < 0.01) compared to women without obesity. In contrast, the utilisation of all other CPS methods did not differ between women with and without obesity. Logistic regression analyses revealed neither an association between CPS behaviour and WBI nor perceived weight bias. Instead, previous cancer diagnoses and knowledge about CPS forms were found to reinforce CPS behaviour.

**Conclusion:**

Although data did not suggest that internalised or perceived weight bias deter women with obesity from undergoing CPS, the role of weight bias has not yet been conclusively clarified. Future studies should address potential methodological limitations and evaluate the effectiveness of most recently established cancer prevention programs and in particular how they affect CPS behaviour in women with obesity.

**Supplementary Information:**

The online version contains supplementary material available at 10.1186/s12905-022-02085-2.

## Background

People with obesity are at higher risk for numerous comorbidities, such as certain forms of cancers [1]. As especially women are affected by the most severe forms of obesity, the risk for incident cancer is more pronounced in women. For example, there is strong evidence that obesity is associated with endometrial cancer, which triples its risk for women who are not of normal weight [2]. Up to 45% of cases of endometrial cancer can be attributed to overweight or obesity in Europe [3]. In addition, there is some evidence to suggest that obesity is also associated with ovarian and cervix cancer [2]. Moreover, death from cancers in the ovary, uterus, cervix, and breast are particularly associated with higher BMI or type 2 diabetes mellitus (T2DM) [4–6].

In consideration of the increased risk for women with obesity to develop gynaecological forms of cancer, the importance of cancer prevention screenings (CPS) becomes obvious. These screenings are examined to detect cancer in the early stages and shall thus facilitate the avoidance of cancer development by removing pre-cancer tissue.

In Germany, all standard CPS (i.e., vaccination, Pap, clinical breast examination, mammograms, faecal occult blood tests (FOBT), and colonoscopy) are offered (staggered by age) and fully covered by statutory and private health insurances as displayed in Table 1.

**Table 1:** Recommended guidelines for CPS according to the German Cancer Research Centre (all fully financed by statutory and private health insurance)

Cancer prevention (CP) services	Recommendations
*Cervical CP*^1^	
Human papillomavirus (HPV) vaccination conducted by gynaecologists	Girls aged 9–17 up to three single vaccinations
Papanicolaou (Pap) smear test conducted by gynaecologists	Annual Pap smear test for women aged 20 and above *Since 2020 (after data collection) Pap smear and HPV test every three years for women aged 35 and above*
*Breast CP*	
Examination of the breast conducted by gynaecologists	Annual clinical breast examination for women aged 30 and above
Mammography screening conducted in qualified medical facilities	Mammogram (X-ray examination) every two years for women aged 50–69
*Colorectal CP*	
Faecal occult blood test (FOBT)	Annual FOBT for women aged 50–54 FOBT every other year for women aged 55 and above
Colonoscopy conducted by proctologists	Colonoscopy every ten years for women aged 55

^1^The recommendations for cervical CPS that are listed above were up-to-date at the time of data assessment but were partly updated in 2020 (updates are displayed in italics). *Source*: German Cancer Research Centre [32]

Although women with obesity are at higher risk for gynaecological forms of cancer, it has been documented that this vulnerable group is less likely to attend gynaecological cancer prevention. A review investigating the association between weight status and screening behaviour for breast, cervical, and colorectal cancer in the US [7] revealed a consistent association between obesity and a decreased utilisation of cervical cancer screenings. The authors found a decreased use of breast cancer screenings among white women with obesity but not among black women [7]. A meta-analysis summarizing six representative studies from the United States on the utilisation of mammography screenings revealed an inverse relationship between increasing BMI and cancer screening behaviour [8]. Concerning colorectal cancer screening, mixed results were found with some studies indicating an association between obesity and reduced screening behaviour whereas other studies reported no effect [7].

When individuals avoid or delay cancer prevention screenings, early-stage cancers remain unnoticed. Detecting cancer diseases in advanced stages makes treatment more difficult. Against this background, decreased health care and prevention-seeking becomes particularly detrimental [9]. As one reason for avoiding or delaying cancer prevention screenings, (perceived) weight bias must be considered a major barrier to health care utilisation [9]. Studies have shown how factors such as disrespectful treatment or negative attitudes among health care providers (HCP), and unsolicited comments about weight loss by HCP hinder women with obesity to undergo health care services [9]. The term weight bias describes how people ascribe negative stereotypes such as being lazy or weak-willed to people with obesity. Obesity can therefore be viewed as a stigmatised condition that leads to negative reactions and behaviour toward those concerned. These anti-fat attitudes stem from the perception that weight is under the individual’s control and that obesity is hence a self-inflicted condition. Although numerous factors have already been identified that lead to a positive energy balance and therefore cause overweight and obesity (e.g., genetics, hormones, medication, socio-cultural and other factors) [10], weight bias is still persistent.

The misperception of obesity as a self-inflicted condition does not only taint the general population’s attitudes and behaviours toward people with obesity [11] but also the attitudes and treatment by health care professionals [12–15], including gynaecologists [16]. People with obesity are confronted with weight bias in numerous areas of life [15]. Since people with obesity are surrounded by weight bias, they are likely to internalise the negative stereotypes and the blame that come along with obesity. Thus, not only perceived weight bias but also weight bias internalisation (WBI) might be considered a barrier that keeps people with obesity from seeking health care and cancer prevention screenings” [9]. This study sought to investigate the difference in screening behaviour of women with and without obesity and the role of perceived weight bias and weight bias internalisation. It is hypothesized that WBI and experiences of weight-based discrimination in the health care setting are associated with lower screening rates in women with obesity.

## Methods

### Aim

This study aims at investigating differences in cancer prevention screening between females with and without obesity. Furthermore, it aims to examine factors that might influence screening behaviour in women with obesity.

### Study design

A population-based cross-sectional survey was conducted with the help of forsa, a large research institute in Germany. It was aimed to assess data of n = 500 females with obesity and n = 500 females without obesity in the general public. Participants took part in telephone interviews lasting about 20 min. The interviews were conducted between September and October 2018.

### Sample and recruitment

The target population comprises of German-speaking females (with and without obesity) aged 25–65 living in Germany, whereby participants were selected through a stratified multilevel random sampling method. Since there is a higher proportion of non-obese females in Germany, the obese sample was increased to attain equal sample sizes of both weight groups (i.e., non-obese and obese). Therefore, forsa conducted a pre-sampling through their daily survey of the German population and assessed the height and weight of female participants who complied with the target population. Based on participants’ reported body measurements, the BMI was calculated using the formula kg/m^2^. Participants who reported a BMI equal to or higher than 30 kg/m^2^ were considered obese, whereas participants with a BMI below 30 kg/m^2^ were classified as non-obese. Participants who belonged to the target population were asked to participate in the study and to give their consent for further contact. To ensure comparability between the obese and non-obese groups, a weighting variable was computed to address differences in age and education.

In total, data of 1003 adult women living in Germany were gathered, of which 503 women reported being non-obese (50.15%, *M* = 24.36, *SD* = 3.27) and 500 women reported a BMI equal to or higher than 30 kg/m^2^ (*M* = 35.10, *SD* = 4.92).

### Instruments

For this study, we compiled a questionnaire which is attached as a Additional file 1. In the following, all relevant items that have been used are described in detail.

### Cancer screening behaviour

In this study, screening behaviour for breast, cervical, and colorectal cancer was assessed by asking participants whether and how often they used certain types of CPS appropriate for their age group. For the analyses, we constructed dichotomous variables that display either insufficient (= 0) or sufficient (= 1) utilisation of CPS, whereas the classification was based on German statutory recommendations. Moreover, we assessed whether and how often participants would examine their breasts themselves. Since there are no guidelines regarding breast self-examination, we refrained from dichotomising this variable and instead operationalised it as ordinal. Table 2 shows how CPS was assessed and how variables were constructed.

**Table 2:** Assessment of cancer prevention screening behaviour

CPS	Sample	Assessment	Construction of variables assessing CPS^1^
HPV vaccination^2^	Women aged < 31	“Have you had an HPV vaccination?” 0 = no, 1 = yes	0 = no vaccination
			1 = being vaccinated
Pap smear test	Women aged ≥ 20	“How often do you use this particular CPS?” five-point Likert scale (0 = never, 1 = less than once a year, 2 = once a year, 3 = twice a year, 4 = more than twice a year)	0 = less than once a year
			1 = at least once a year
Clinical examination of the breast	Women aged ≥ 30		
Mammography screening	Women aged 50–69	“Have you ever used this particular CPS and if so how often?” seven-point Likert scale (0 = never, 1 once, 2 = twice, 3 = three times, 4 = four times, 5 = five times, 6 = more than five times)	0 = less than every two years
			1 = at least every two years
FOBT	Women aged ≥ 50		0 = less than once a year for women aged 50–54 or less than every other year for women aged ≥ 55
			1 = at least annually for women aged 50–54 or at least every other year for women aged ≥ 55
Colonoscopy	Women aged ≥ 50		0 = no colonoscopy or less than every ten years
			1 = at least every ten years
Self-examination of the breast	Women aged ≥ 30	“How often do you examine your breast by yourself?” Seven-point Likert scale (0 = never, 1 = infrequent, 2 = once a month, 3 = several times a month, 4 = once a week, 5 = several times a week, 6 = daily)	Metric variable

^1^The value 0 displays insufficient, the value of 1 displays at least sufficient utilisation of CPS

^2^The HPV vaccination is a relatively new prevention method for cervical cancer that is applied since the early 2000s. We therefore asked only participants under 31 years if they had been HPV vaccinated

### Weight status

At the time of recruitment, participants were asked to state their body weight and height. The Body Mass Index (BMI) was calculated afterwards, whereas participants with a BMI equal to or higher than 30 kg/m^2^ were considered obese, and participants with a BMI lower than 30 kg/m^2^ were considered non-obese.

Moreover, we assessed the participants' self-perceived weight status. We asked them to describe their weight status by choosing the best matching category (i.e., extremely underweight, underweight, slightly underweight, normal weight, slightly overweight, overweight, obese). For logistic regression analyses among women with obesity (Table 6), we compared categories (e.g., slightly overweight, overweight, obese) with the reference category of not overweight (subsuming all remaining categories).

### Experienced weight bias

As a measure of experienced weight bias, we first asked participants whether they have ever perceived the treatment of practitioners of different disciplines (i.e., physicians, gynaecologists, proctologists, dermatologists, dentists, and orthopaedists) as inadequate. We calculated a dichotomous variable with the value of 0 indicating that participants have perceived the treatment of health care professionals of all aforementioned disciplines as adequate, whereas the value of 1 indicates that the treatment of at least one HCP was perceived as inadequate. Second, women with obesity were asked whether they had, in general, ever experienced weight-based discrimination, which is displayed in a dichotomous variable (0 = no, 1 = yes). Third, participants with obesity were asked whether they have ever experienced weight-based discrimination by HCPs. Again, a dichotomous variable was calculated (0 = no experienced weight-based discrimination of HCP, 1 = experienced weight-based discrimination by at least one HCP). Moreover, we asked women with obesity if any medical procedure has been refused because of their weight (0 = no, 1 = yes).

### Internalised weight bias

The internalisation of weight bias was measured by using the Weight Bias Internalisation Scale (WBIS) developed by Durso and colleagues [17]. The WBIS is one of the most used instruments that measures to what extent people with obesity have internalised negative attitudes and stereotypes and apply these stereotypes to themselves. The WBIS consists of 11 items (e.g., ‘‘I hate myself for being overweight’’) that are supposed to be rated on a seven-point Likert scale ranging from 1 (“strongly disagree”) to 7 (“strongly agree”). For the German version of the WBIS, one item was excluded because it was negatively correlated with the remaining items (“As an overweight person, I feel that I am just as competent as anyone”) [18]. For analysis, a sum score of the remaining 10 items was calculated, whereby higher scores indicated a stronger internalisation of weight bias. A reliability test revealed a Cronbach’s alpha of 0.85 and therefore good reliability. In this study, participants with obesity reported WBI ranging from 10 to 70 (M = 29.40, SD = 12.43).

### Cancer awareness

To assess cancer awareness, participants were asked whether they have ever been diagnosed with cancer. If participants answered yes, we enquired about the type of cancer. Furthermore, we asked if cancer diagnoses in their close environment (i.e., family members, friends, or colleagues) were known. We also enquired about participants’ knowledge of recommended CPS for their respective age group and created a dichotomous variable based on their answers (0 = participants had no knowledge about CPS recommendations, 1 = participants knew about CPS recommendations for their age group).

### Confounding variables

Participants were asked to provide information regarding their highest educational degree. For the analysis, a dichotomous variable representing educational attainment was generated with a value of 1 indicating at least 12 years in school and a value of 0 indicating less than 12 years in school. Participants who reported to still be in school (n = 2) or stated to have “other degree” (n = 10) were excluded because these categories could not be classified to be more or less 12 years of education. We gathered another sociodemographic variable related to participants’ net household income per month. This variable was assessed with ten response categories with a range of 500 Euro each. Based on these data, a variable reporting net household income per capita was generated and divided into four groups (quartiles). For logistic regression models, we included income in quartiles as a confounding variable and reported results regarding the first quartile (lowest income group).

We also assessed participants’ form of health care insurance (i.e., private or statutory), since CPS behaviour might differ between people who are privately insured compared to those covered by statutory health insurance.

### Statistical analysis

All analyses were run with STATA 15.1 [20]. First, we conducted chi-square tests to investigate differences in the cancer screening behaviour of women with and without obesity. For this, the frequency of using single CPS methods was compared between weight groups.

In a second step, logistic regression models were calculated to assess determining factors of CPS behaviour in women with and without obesity. For this, we conducted logistic regression analyses that investigated determining factors in the full sample (women with and without obesity) and in the sample of women with obesity only. We conducted four logistic regression models, with the utilisation of Pap smear tests, clinical breast examinations, FOBT, and colonoscopies (0 = utilisation less frequently than recommended, 1 = utilisation at least as frequently as recommended) as respective outcome variables. We refrained from conducting a logistic regression analysis to determine the promoting or suppressing variables of HPV vaccination because of the small sample size of women who had been vaccinated (n = 27).

In the full sample analyses, we included as explanatory variables the weight group (0 = non-obese, 1 = obese) as well as cancer awareness (i.e., cancer diagnoses in participants and their close social environment as well as knowledge about CPS). In the sample containing only women with obesity, we included variables assessing cancer awareness, self-perceived weight, WBI, and experienced weight bias (i.e., general weight-based discrimination, weight-based discrimination by HCP, and insufficient treatment by HCP) as explanatory factors. Participants’ sociodemographic characteristics (i.e., type of health care insurance, age, education, income) were included as confounding variables in all models.

Since women who are diagnosed with breast or colorectal cancers need to undergo diagnostic procedures that might be misclassified as cancer *prevention* screenings, these women were excluded from the respective models. In our sample, only one participant stated to be currently diagnosed with colorectal cancer, whereas n = 5 participants were at the time of data assessment diagnosed with breast cancer. We excluded these participants to avoid such misclassifications. All analyses were only conducted for the women who were in the CPS’ target group (according to their age; see Table 2) and who reported complete data.

## Results

Overall, data from 1003 women were analysed. Participants’ detailed characteristics and other descriptive variables are shown in Table 3.

**Table 3:** Participants’ characteristics

	Total (n = 1003)	BMI < 30 (n = 503)	BMI ≥ 30 (n = 500)	Sample BMI < 30 vs. sample BMI ≥ 30
	n (%)	n (%)	n (%)	Chi^2^, N, *p*-value, and Cramer’s V
*Basic sociodemographic characteristics*				
Education				χ^2^ (1, n = 1003) = 20.4299, *p* < 0.001, Cramer’s V = − 0.1427
< 12 years in school	466 (46.46%)	198 (39.36%)	268 (53.60%)	
≥ 12 years in school	537 (53.54%)	305 (60.64%)	232 (46.40%)	
Age				χ^2^ (3, n = 1003) = 0.7829, *p* = 0.854, Cramer’s V = 0.0279
25–34	70 (6.98%)	36 (7.16%)	34 (6.80%)	
35–44	128 (12.76%)	65 (12.92%)	63 (12.60%)	
45–54	283 (28.22%)	147 (29.22%)	136 (27.20%)	
55–65	522 (52.04%)	255 (50.70%)	267 (53.40%)	
Mean age (SD)	52.75 (9.72)	52.6 (9.73)	52.89 (9.71)	
Household income in Euro^1^				χ^2^ (9, n = 912) = 36.9948, *p* < 0.001, Cramer’s V = 0.2014
< 500	9 (1.0%)	6 (1.4%)	3 (0.6%)	
500 < 1000	61 (6.7%)	13 (3.0%)	48 (10.1%)	
1000 < 1500	90 (9.9%)	37 (8.6%)	53 (11.2%)	
1500 < 2000	124 (13.6%)	61 (13.9%)	63 (13.3%)	
2000 < 2500	133 (14.6%)	60 (13.7%)	73 (15.4%)	
2500 < 3000	92 (10.1%)	37 (8.5%)	55 (11.6%)	
3000 < 3500	99 (10.7%)	46 (10.5%)	53 (11.2%)	
3500 < 4000	80 (8.8%)	48 (10.9%)	32 (6.8%)	
4000 < 4500	78 (8.6%)	44 (10.1%)	34 (7.2%)	
≥ 4500	146 (16.0%)	86 (19.6%)	60 (12.7%)	
Health insurance				χ^2^ (1, n = 1002) = 6.8299, *p* = 0.009, Cramer’s V = − 0.0826
Statutory health insurance	119 (88.1%)	429 (85.5%)	454 (90.8%)	
Private health insurance	883 (11.9%)	73 (14.5%)	46 (9.2%)	
*Weight status*				
BMI				
Underweight (BMI < 18.5)	13 (1.3%)	13 (1.3%)	–	
Normal weight (18.5 ≥ BMI < 25)	268 (26.72%)	268 (26.72%)	–	
Overweight (25 ≥ BMI < 30)	222 (22.13%)	222 (22.13%)	–	
Nonobese (BMI < 30)	503 (50.15%)	503 (50.15%)	–	
Obesity (BMI ≥ 30)	500 (49.85%)	–	500 (49.85%)	
Obesity class I (30 ≥ BMI < 35)	301 (30.01%)	–	301 (30.01%)	
Obesity class II (35 ≥ BMI < 40)	129 (12.86%)	–	129 (12.86%)	
Obesity class III (BMI ≥ 40)	70 (6.98%)	–	70 (6.98%)	
Mean BMI (SD)	29.72 (6.80)	24.36 (3.27)	35.10 (4.92)	
Self-perceived weight status				
Extremely underweight	3 (0.3%)	3 (0.6%)	–	
Underweight	4 (0.4%)	4 (0.8%)	–	
Slightly underweight	26 (2.6%)	26 (5.17%)	–	
Normal weight	252 (25.12%)	245 (48.71%)	7 (1.40%)	
Slightly overweight	212 (21.14%)	154 (30.62%)	58 (11.60%)	
Overweight	312 (31.11%)	68 (13.52%)	244 (48.80%)	
Obese	194 (19.34%)	3 (0.6%)	191 (38.20%)	
*Experiences with HCPs*				
Inadequate treatment by HCPs				χ^2^ (1, n = 1003) = 4.6272, *p* = 0.031, Cramer’s V = − 0.0679
Experienced	674 (67.2%)	354 (70.4%)	320 (64.0%)	
Not experienced	329 (32.8%)	149 (29.6%)	180 (36.0%)	
*Weight bias*				
WBI				
Q1 WBIS (Score 10 ≤ 20)	–	–	142 (28.4%)	
Q2 WBIS (Score 20 ≤ 27)	–	–	114 (22.8%)	
Q3 WBIS (Score 27 ≤ 37)	–	–	127 (25.4%)	
Q4 WBIS (Score 37 ≤ 70)	–	–	117 (23.4%)	
Mean WBIS (SD)	–	–	29.40 (12.43)	
General weight-based discrimination				–
Yes	–	–	186 (37.2%)	
No	–	–	314 (62.8%)	
Weight-based discrimination by HPCs				–
Yes	–	–	138 (27.6%)	
No	–	–	362 (72.4%)	
Treatment refused because of weight				–
Yes	–	–	14 (2.8%)	
No	–	–	486 (97.2%)	
*Experiences with cancer*				
Current/previous cancer diagnosis in participants				χ^2^ (1, n = 1002) = 0.3369, *p* = 0.562, Cramer’s V = − 0.0183
Yes	87 (8.7%)	41 (8.2%)	46 (9.2%)	
No	915 (91.3%)	461 (91.8%)	454 (90.8%)	
Current/previous cancer in participant’s environment				χ^2^ (1, n = 1001) = 0.0769, *p* = 0.781, Cramer’s V = − 0.0088
Yes	772 (77.1%)	389 (77.5%)	383 (76.8%)	
No	229 (22.9%)	113 (22.5%)	116 (23.2%)	
*Knowledge about CPS*				
Knowledge about Pap smear (women aged 25–65)				χ^2^ (1, n = 1003) = 2.7962, *p* = 0.094, Cramer’s V = − 0.0528
Yes	550 (54.8%)	289 (57.5%)	261 (52.2%)	
No	453 (45.2%)	214 (42.5%)	239 (47.8%)	
Knowledge about HPV vaccination (women aged ≤ 30)				χ^2^ (1, n = 32) = 3.1643, *p* = 0.075, Cramer’s V = − 0.3145
Yes	27 (84.4%)	17 (94.4%)	10 (71.4%)	
No	5 (15.6%)	1 (5.6%)	4 (28.6%)	
Knowledge about clinical breast examination (women aged 30–65)				χ^2^ (1, n = 985) = 0.091, *p* = 0.754, Cramer’s V = − 0.0100
Yes	182 (18.5%)	93 (18.9%)	89 (18.1%)	
No	803 (81.5%)	400 (81.1%)	403 (81.9%)	
Knowledge about mammography (women aged 50–65)				χ^2^ (1, n = 711) = 0.4475, *p* = 0.504, Cramer’s V = − 0.0251
Yes	381 (53.6%)	192 (54.9%)	189 (52.3%)	
No	330 (46.4%)	158 (45.1%)	172 (47.7%)	
Knowledge about faecal occult blood test (FOBT) (women aged 50–65)				χ^2^ (1, n = 711) = 1.3664, *p* = 0.242, Cramer’s V = − 0.0438
Yes	219 (30.8%)	115 (32.9%)	104 (28.8%)	
No	492 (69.2%)	235 (67.1%)	257 (71.2%)	
Knowledge about colonoscopy (women aged 55–65)				χ^2^ (1, n = 711) = 0.0220, *p* = 0.882, Cramer’s V = − 0.0056
Yes	386 (54.3%)	191 (54.6%)	195 (54.0%)	
No	325 (45.7%)	159 (45.4%)	166 (46.0%)	

^1^n = 912 due to missing values

### Differences in cancer screening behaviour of women with and without obesity

To investigate if there are differences in the frequency of cancer prevention screening of women with and without obesity, chi-square tests were conducted and displayed in Table 4.

**Table 4:** Results of chi^2^ test analysing differences in cancer screening behaviour among weight groups

Cancer screening	Women without obesity	Women with obesity	Chi^2^, N, *p*-value, and Cramer’s V
*Cervical cancer*			
Pap smear (women aged 25–65)			
Never	42 (8.4%)	63 (12.6%)	χ^2^ (4, n = 1002) = 15.08, *p* = 0.005, Cramer’s V = 0.1227
Less than once a year	65 (12.9%)	95 (19.0%)	
Once a year	322 (64.0%)	273 (54.7%)	
Twice a year	68 (13.5%)	59 (11.8%)	
> 2 a year	6 (1.2%)	9 (1.8%)	
Less than recommended	107 (21.3%)	158 (31.7%)	χ^2^ (1, n = 1002) = 13.90, *p* < 0.001, Cramer’s V = − 0.1178
At least as recommended	396 (78.7%)	341 (68.3%)	
HPV vaccination (women aged ≤ 30), who knew about the vaccination			
No	12 (70.6%)	7 (70.0%)	χ^2^ (1, n = 27) = 0.001, *p* = 0.974, Cramer’s V = 0.0062
Yes	5 (29.4%)	3 (30.0%)	
*Breast cancer*			
Clinical breast examination (women aged 30–65)			
Never	36 (7.3%)	56 (11.5%)	χ^2^ (4, n = 982) = 14.41, *p* = 0.006, Cramer’s V = 0.1211
Less than once a year	59 (11.9%)	89 (18.2%)	
Once a year	314 (63.7%)	274 (56.0%)	
Twice a year	73 (14.8%)	61 (12.5%)	
> 2 a year	11 (2.2%)	9 (1.8%)	
Less than recommended	95 (19.3%)	145 (29.7%)	χ^2^ (1, n = 982) = 14.33, *p* < 0.001, Cramer’s V = − 0.1208
At least as recommended	398 (80.7%)	344 (70.3%)	
Self-examination of the breast (women aged 25–65)			
Never	114 (27.6%)	103 (25.7%)	χ^2^ (6, n = 814) = 12.67, *p* = 0.056, Cramer’s V = 0.1228
Less than once a month	90 (21.8%)	65 (16.2%)	
Once a month	93 (22.5%)	92 (22.9%)	
Several times a month	35 (8.5%)	27 (6.7%)	
Once a week	44 (10.7%)	60 (14.9%)	
Several times a week	19 (4.6%)	34 (8.5%)	
Daily	18 (4.4%)	20 (5.0%)	
Mammography (women aged 50–65)			
Not sufficient	170 (48.6%)	165 (45.8%)	χ^2^ (1, n = 710) = 0.53, *p* = 0.465, Cramer’s V = 0.0276
Sufficient	180 (51.4%)	195 (54.2%)	
*Colorectal cancer*			
Faecal occult blood test (FOBT) (women aged 50–65)			
Not sufficient	251 (72.1%)	245 (69.2%)	χ^2^ (1, n = 702) = 0.72, *p* = 0.396, Cramer’s V = 0.0320
Sufficient	97 (27.9%)	109 (30.8%)	
Colonoscopy (women aged 55–65)			
Not sufficient	102 (40.0%)	129 (48.5%)	χ^2^ (1, n = 521) = 3.81 *p* = 0.051 Cramer's V = − 0.0855
Sufficient	153 (60.0%)	137 (51.5%)	

### Cervical cancer screenings

The Chi-square test revealed differing screening behaviour between women with and without obesity (χ^2^(4) = 15.08, *p* = 0.005, n = 1002). These findings indicate that women with obesity use Pap smear test less often compared to women without obesity. Moreover, we analysed differences in the utilisation of HPV vaccination between women with and without obesity. The Chi-square test revealed no significant differences between weight groups (χ^2^(1) = 0.001, *p* = 0.974, n = 27).

### Breast cancer screenings

Differences in the utilisation of clinical breast examinations as prevention screening for breast cancer across weight groups were examined for women aged 30 or older. A chi-square test indicates a less frequent utilisation of clinical breast examination for women with obesity (χ^2^(4) = 14.41, *p* = 0.006, n = 982). However, women of different degrees of obesity did not differ in their frequency of clinical breast examinations (χ^2^(2) = 0.36, *p* = 0.834, n = 489). Neither did we find differences in women with and without obesity regarding how often they themselves examine their breasts for lumps (χ^2^(6) = 12.67, *p* = 0.056, n = 814; Table 1) nor did we find group differences regarding the frequency of mammography screenings (χ^2^(1) = 0.53, *p* = 0.465, n = 710).

### Colorectal cancer screenings

Chi-square testing neither revealed differences between women with and without obesity regarding the utilisation of FOBT (χ^2^(1) = 0.72, *p* = 0.396, n = 702) nor differences in the utilisation of colonoscopy (χ^2^ (1) = 3.81 *p* = 0.051, n = 522).

### Determinants of the utilisation of cancer prevention screenings

The results of the logistic regression models assessing influencing factors for CPS behaviour in women with and without obesity are reported in Table 5 and the results for women with obesity only are reported in Table 6.

**Table 5:** Logistic regression model: influencing factors on the utilisation of clinical cancer prevention screenings among women with and without obesity

	Pap smear (women aged ≥ 20			Clinical breast examination (women aged ≥ 30)			Mammography* (women aged ≥ 50)			Faecal occult blood test* (women aged ≥ 50)			Colonoscopy* (women aged ≥ 55)		
	OR	*p*	[95% CI]	OR	*p*	[95% CI]	OR	*p*	[95% CI	OR	*p*	[95% CI	OR	*p*	95% CI
Weight Status^1^	0.63**	**.004**	**(0.46–0.86)**	0.61**	**.003**	**(0.44–0.85)**	1.11	.564	(0.78–1.58)	1.28	.189	(0.89–1.85)	0.67	.056	(0.44–1.01)
Cancer awareness															
Current/previous (other) cancer diagnosis in participants^2^	2.34**	**.008**	**(1.24–4.39)**	2.20*	**.017**	**(1.15–4.21)**	3.84***	** < .001**	**(2.09–7.05)**	0.78	.402	(0.43–1.40)	2.83**	**0.003**	**(1.41–5.66)**
Current/previous (other) cancer in participant’s environment^3^	1.19	.336	(0.83–1.71**)**	1.35	.109	(0.94–1.95)	1.36	.134	(0.91–2.04)	1.25	.325	(0.80–1.93)	1.64*	**.039**	**(1.03–2.63)**
CPS knowledge^4^	2.26***	** < .001**	**(1.65–3.10)**	2.35***	**.001**	**(1.44–3.85)**	1.39	.066	(0.98–1.97)	1.55*	**.023**	**(1.06–2.27)**	4.05***	** < .001**	**(2.68–6.13)**
Confounding variables															
Health Insurance^5^	1.86*	**.043**	**(1.02–3.41)**	2.06*	**.035**	**(1.05–4.04)**	0.72	.259	(0.41–1.27)	2.10**	**.010**	**(1.20–3.70)**	2.27*	**.018**	**(1.15–4.49)**
Age	0.97***	< **.001**	**(0.95–0.99)**	0.98**	**.009**	**(0.96–0.99)**	0.84***	** < .001**	(**0.81–0.88)**	0.87***	** < .001**	**(0.84–0.91)**	1.14***	** < .001**	**(1.07–1.22)**
Educational Level^6^	0.81	.256	(0.58–1.12)	0.81	.222	(0.58–1.13)	.82	.280	(0.57–1.18)	1.01	.964	(0.69–1.47)	0.98	.945	(0.64–1.51)
Marital status^7^	1.48*	**.013**	**(1.09–2.02)**	**1.55**	**.002**	**(1.13–2.13)**	1.13	.476	(0.80–1.61)	1.15	.441	(0.80–1.66)	1.24	.301	(0.82–1.86)
Household income^8^															
2. Quartile	1.27	.186	(0.84–1.93)	1.55*	**.039**	**(1.08–2.47)**	1.19	.481	(0.73–1.95)	1.25	.409	(0.74–2.12)	1.12	.713	(0.62–2.01)
3. Quartile	1.25	.264	(0.80–1.95)	1.53	.063	(1.02–2.48)	0.98	.921	(0.59–1.60)	1.62	.074	(0.95–2.76)	1.27	.419	(0.71–2.29)
4. Quartile	1.14	.546	(0.73–1.78)	1.94**	**.006**	**(1.21–3.09)**	1.12	.660	(0.68–1.82)	1.55	.104	(0.91–2.61)	1.02	.947	(0.58–1.79)
n	910			891			642			638			475		
Prob > chi2	< 0.001			< 0.001			< 0.001			< 0.001			< 0.001		
Pseudo R2	0.07			0.06			0.13			0.09			0.15		

Bold indiciates the siginnificant values: ***p ≤ 0.001, **p ≤ 0.01, *p ≤ 0.05

*Women who reported a current breast (n = 5) or colorectal (n = 1) cancer diagnosis were excluded in the corresponding models since diagnostic procedures or interventions could have been misclassified as CPS behaviour. Outcome variable sufficient utilisation cancer screenings (0 = not sufficient, 1 = sufficient); OR Odds ratios

^1^0 = not obese, 1 = obese

^2^^–^^4^0 = no, 1 = yes

^5^0 = statutory health insurance, 1 = private health insurance

^6^0 = less than 12 years of education

^7^0 = not married or not living together, 1 = married and living together

^8^Reference category (= 0): first quartile

**Table 6:** Logistic regression model: influencing factors on the utilisation of clinical cancer prevention screenings among women with obesity

	Pap smear (women with obesity, aged ≥ 20			Clinical breast examination (women with obesity, aged ≥ 30)			Mammography* (women with obesity, aged ≥ 50)			Faecal occult blood test* (women with obesity, aged ≥ 50)			Colonoscopy* (women with obesity, aged ≥ 55)		
	OR	*p*	95% CI	OR	*p*	95% CI	OR	*p*	95% CI	OR	*p*	95% CI	OR	*p*	95% CI
Self-perceived Weight Status^1^															
Slightly overweight	1.39	.730	(0.21–9.12)	1.55	.642	(0.24–9.98)	0.29	.207	(0.04–1.98)	1.76	.635	(0.17–18.00)	1.26	.632	(0.49–3.24)
Overweight	0.74	.737	(0.12–4.38)	0.55	.501	(0.10–3.14)	0.34	.244	(0.05–2.10)	3.24	.300	(0.35–30.01)	1.13	.709	(0.59–2.20)
Extremely overweight	0.49	.438	(0.08–2.96)	0.53	.484	(0.09–3.10)	0.33	.237	(0.05–2.09)	3.53	.272	(0.37–33.32)	Omitted because of collinearity		
Experienced weight bias															
Inadequate treatment by HCPs^2^	0.89	.604	(0.56–1.39)	0.93	.760	(0.58–1.48)	0.96	.938	(0.57–1.68)	1.34	.315	(0.76–2.36)	1.08	.810	(0.57–2.06)
Treatment refused^3^	0.88	.842	(0.24–3.16)	0.54	.316	(0.16–1.81)	0.71	.726	(0.11–4.67)	0.87	.867	(0.17–4.53)	0.79	.860	(0.06–10.70)
Weight-based discrimination by HPCs^4^	1.03	.894	(0.62–1.72)	0.95	.860	(0.56–1.61)	1.04	.914	(0.55–1.94)	0.83	.566	(0.44–1.56)	1.60	.208	(0.77–3.33)
General weight-based discimination^5^	1.04	.887	(0.64–1.67)	1.04	.864	(0.64–1.71)	1.21	.538	(0.66–2.20)	0.96	.889	(0.52–1.75)	0.77	.478	(0.38–1.57)
Internalised weight bias															
WBIS	0.99	.520	(0.98–1.01)	1.01	.518	(0.99–1.02)	0.99	.174	(0.96–1.01)	1.00	.885	(0.98–1.02)	1.05***	**.001**	**(1.02–1.08)**
Cancer awareness															
Current/previous (other) cancer diagnosis in participants^6^	2.43*	**.036**	**(1.06–5.58)**	1.78	.160	(0.80–3.95)	3.93**	**.002**	**1.62–9.55)**	0.63	.290	(0.27–1.48)	3.62*	**.012**	**(1.32–9.92)**
Current/previous (other) cancer in participant’s environment^7^	1.11	.647	(0.69–1.84)	1.31	.291	(0.79–2.18)	1.64	.169	(0.86–2.37)	1.07	.827	(0.57–2.04)	1.61	.166	(0.82–3.17)
CPS knowledge^8^	2.17***	** < .001**	**(1.43–3.30)**	3.33***	**.001**	**(1.66–6.66)**	1.43	.169	(0.86–2.37)	1.45	.205	(0.82–2.55)	3.84***	** < .001**	**(2.10–7.02)**
Confounding variables															
Health Insurance^9^	1.39	.452	(0.59–3.32)	1.98	.174	(0.74–5.27)	0.90	.809	(0.37–2.18)	2.03	.120	(0.83–4.94)	2.18	.142	(0.77–6.16)
Age	0.98*	**.033**	**(0.95–1.00)**	0.99	.294	(0.96–1.01)	0.80***	** < .001**	**(0.75–0.86)**	0.84***	** < .001**	**(0.79–0.89)**	1.13*	**.012**	**(1.03–1.24)**
Educational Level^10^	1.04	.873	(0.67–1.60)	0.98	.940	(0.63–1.54)	1.03	.926	(0.60–1.75)	1.24	.430	(0.72–2.13)	1.33	.376	(0.71–2.50)
Marital Status^11^	**1.55**	**.040**	**(1.02–2.35)**	1.70	.016	(1.11–2.62)	1.18	.515	(0.54–2.36)	0.94	.827	)0.56–1.59)	1.23	.497	(0.41–2.16)
Household income^12^															
2. Quartile	1.05	.860	(0.62–1.78	1.69	.060	(1.07–3.13)	0.94	.865	(0.47–1.88)	1.03	.925	(0.50–2.12)	1.00	.998	(0.45–2.23)
3. Quartile	1.43	.249	(0.78–2.64	1.78	.067	(1.04–3.49)	1.10	.789	(0.53–2.29)	2.10*	**.046**	**(1.01–4.36)**	1.72	.216	(0.73–4.05)
4. Quartile	1.21	.552	(0.65–2.26	1.93*	**.049**	**(0.98–3.58)**	1.13	.739	(0.54–2.36)	1.00	.990	(0.46–2.19)	0.94	.876	(0.41–2.16)
n	**473**			**463**			**339**			**336**			**247**		
Prob > chi^2^	**0.003**			**0.001**			** < 0.001**			** < 0.001**			** < 0.001**		
Pseudo R^2^	**0.065**			**0.075**			**0.185**			**0.145**			**0.170**		

Bold indiciates the siginnificant values: ***p ≤ 0.001, **p ≤ 0.01, *p ≤ 0.05

*Women who reported a current breast (n = 5) or colorectal (n = 1) cancer diagnosis were excluded in the corresponding models since diagnostic procedures or interventions could have been misclassified as CPS behaviour; Outcome variable sufficient utilisation cancer screenings (0 = not sufficient, 1 = sufficient); OR Odds ratios; HCP = Health care professional; CPS = Cancer prevention screening

^1^Reference category (= 0): not overweight

^2^^–^^8^0 = no, 1 = yes

^9^0 = statutory health insurance, 1 = private health insurance

^10^0 = less than 12 years of education

^11^0 = not married or not living together, 1 = married and living together

^12^Reference category (= 0): first quartile

***p ≤ 0.001, **p ≤ 0.01, *p ≤ 0.05; Insignificant variables were excluded (i.e., experiences with cancer in participants’ environment)

### Determinants of cancer prevention screening behaviour among women with different weight status

As Chi-square tests already indicated, women with and without obesity differ in their use of cervical cancer screenings (i.e., Pap test, OR 0.62, *p* = 0.003) and clinical breast examinations (OR 0.60, *p* = 0.002). In addition, results regarding the utilisation of colonoscopy also indicate a difference between women with and without obesity. However, these results were not significant (OR 0.66, *p* = 0.051).

A current or previous cancer diagnosis in women increased the chances to undergo pap tests (OR 2.34, *p* = 0.008), clinical breast examinations (OR 2.20, *p* = 0.017), mammography screenings (OR 2.84, *p* < 0.001), and colonoscopies (OR 2.83, *p* = 0.003), but not FOBT screenings (OR 0.78, *p* = 0.402). Knowing someone who has a previous or current cancer diagnosis in their close social environment increased the chance to undergo colonoscopy as recommended (OR 1.64, *p* = 0.039). Having knowledge about the respective CPS increased the chance to utilise Pap tests (OR 2.26, *p* < 0.001), clinical breast examinations (OR 2.35, *p* = 0.001), FOBT (OR 1.55, *p* = 0.23), and colonoscopies (OR 4.05, *p* < 0.001) as recommended. Data indicate that knowledge about mammography screenings also increases the likelihood to undergo this CPS as recommended. However, this result was not significant (OR 1.39, *p* = 0.66).

Having private health insurance increased the chance to undergo Pap tests (OR 1.86, *p* = 0.043), clinical breast examinations (OR 2.06, *p* = 0.035), FOBT (OR 2.10, *p* = 0.010), and colonoscopies (OR 2.27, *p* = 0.018) as recommended. Higher age was associated with a greater risk of not making use of Pap tests (OR 0.97, *p* < 0.001), clinical breast examinations (OR 0.98, *p* = 0.009), as well as mammography (OR 0.84, *p* < 0.001) and FOBT (OR 0.87, *p* < 0.001) screenings. In contrast, higher age increased the likelihood to undergo colonoscopies as recommended (OR 1.14, *p* < 0.001).

Participants’ educational degree was not related to any CPS behaviour, whereas participants’ household income was positively related to clinical breast examinations. Participants who had more income compared to their less affluent counterparts were more likely to undergo clinical breast examinations (e.g., 2nd quartile: OR 1.55, *p* = 0.039; 4th quartile: OR 1.94, *p* = 0.006). Married participants who live with their spouse were more likely to undergo Pap screenings (OR 1.48, *p* = 0.013) and clinical breast examinations (OR 1.55, *p* = 0.002).

### Determinants of cervical cancer screenings in women with obesity

Neither self-perceived weight status nor perceived or internalised weight bias were found to be significantly associated with cervical CPS. However, the logistic regression model revealed that personal experiences with cancer (i.e., previous cancer diagnoses) (OR 2.43, *p* = 0.036) and knowledge about the Pap smear as CPS (OR 2.17, *p* < 0.001) significantly increased the likelihood to undergo Pap smear screenings. Furthermore, higher age was also found to be negatively associated with recommended utilisation of cervical CPS (OR 0.98, *p* = 0.033). Being married and living together with one’s spouse increased the chance to undergo Pap tests as recommended (OR 1.55, *p* = 0.040).

### Determinants of breast cancer screenings in women with obesity

We found no significant association between the utilisation of breast CPS (i.e., clinical breast examinations or mammography screenings) and self-perceived weight status or weight bias. The logistic regression analysis revealed that participants' knowledge about clinical breast examination as CSP significantly increased the chance to annually undergo this form of CPS (OR 3.33, *p* < 0.001). Income was also significantly related to clinical breast examination. The data revealed that the group with the highest income (4th quartile OR 1.93, *p* = 0.049)) are more likely to undergo clinical breast examinations as recommended compared to participants of the lowest income group (1st quartile).

The logistic regression model investigating influencing factors for the utilisation of mammography screenings services revealed that women who were previously diagnosed with cancer (OR 3.93, *p* = 0.002) were more likely to undergo regular mammography screenings. In turn, higher age was negatively associated with mammography utilisation (OR 0.80, *p* < 0.001).

### Determinants of colorectal cancer screenings in women with obesity

Neither self-perceived weight status nor internalised and perceived weight bias were found to determine the utilisation of FOBT and colonoscopies in women with obesity. Instead, logistic regression analysis indicates that women with obesity of a younger age (OR 0.84, *p* < 0.001) were more likely to undergo FOBT as recommended.

We also investigated which factors might influence the utilisation of colonoscopies in women with obesity. The results of logistic regression analysis indicate that women who have stronger internalised weight bias were slightly but significantly more likely to undergo this kind of CPS (OR 1.05, *p* < 0.001). Perceived weight status and weight bias on the other hand were not significantly associated with the utilisation of colonoscopies. Moreover, women who have experienced cancer themselves (OR 3.62, *p* = 0.012), who knew about colonoscopy as CPS (OR 3.84, *p* =  < 0.001), and who were older (OR 1.13, *p* = 0.012) were found to be more likely to undergo colonoscopies.\

## Discussion

In this study, we sought to investigate differences in the utilisation of CPS in women with and without obesity. Previous findings have indicated that women with obesity are less likely to undergo medical screenings, particularly cancer screenings [7]. We conducted this survey because data for Germany were lacking in this regard and influencing factors that might either promote or suppress CPS behaviour in women with obesity have not been investigated in Germany before.

We determined differences in CPS behaviour among women of different weight groups for the utilisation of Pap smears and clinical breast examinations. Almost one-third of women with obesity do not undergo Pap smears (31.7%) and clinical breast examinations (29.7%) as frequently as recommended, whereas only every fifth woman without obesity does not use cervical (21.3%) and breast CPS (19.3%) as recommended. In contrast, we found no differences in breast self-examination between women with and without obesity. This is a rather remarkable outcome because it somehow implies that there are no weight-based differences when CPS does not require a medical assistant but one’s self-responsibility. Women with obesity can thus not be considered less careful.

Interestingly, Pap tests and clinical breast examinations are examined by gynaecologists, whereas we found no significant weight-based differences in the utilisation of CPS that are not examined by gynaecologists, i.e., mammography screenings and faecal occult blood tests. Although the chi-square test (Table 4, χ^2^(1) = 3.81 *p* = 0.051), as well as our logistic regression model (Table 5, OR 0.67, *p* = 0.056), indicate that women with and without obesity differ regarding their utilisation of colonoscopies, these results are statistically not significant.

These findings may be explained by the fact that the HCP that conduct mammograms and colorectal CPS are not necessarily involved in the standard care of patients and are thus not known by patients (that well). Women who might have (un)conscious worries and aversions regarding their gynaecologists or the doctor’s receptionists, or who are displeased about the practice equipment might avoid regular health care screenings.

Furthermore, the findings reflect some degree of the study situation that was reviewed by Aldrich and Hackely [19] who found strong evidence that women with obesity are less likely to undergo cervical cancer screening. Although the review also indicates a decreased use of breast CPS (mammograms) in women with obesity compared to women of normal weight, these findings are not based on strong evidence due to mixed results in the review [19]. The systematic review of Cohen et al. [7] also found that weight-based differences in CPS behaviour were more pronounced in the utilisation of cervical cancer screenings, whereas studies investigating the utilisation of breast cancer between women with and without obesity were found to be less consistent. Cohen et al. also reviewed the adherence to colorectal cancer screenings in women of different weight groups and found a more inconsistent study situation [7].

The effect of obesity on decreased screening compliance often was more pronounced in the highest categories. Factors that might increase or decrease CPS behaviour in women with obesity were investigated in logistic regression models. Although we found that women with obesity are less likely to use some CPS as recommended, we did—contrary to our expectations—not find a significant association between CPS behaviour and obesity-based discrimination or WBI (apart from colonoscopies). It is difficult to explain these results, but it might be related to the fact that the applied questions regarding perceived obesity-related discrimination (“Have you ever felt excluded or discriminated against [by HCP] because of your weight?”) might have been too vague. Aldrich and Hackely, for example, have also discussed negative attitudes, e.g. embarrassment, stress, and fear as potential reasons for differing cancer screening behaviour between women with and without obesity [20]. These specific negative thoughts and emotions that participants associate with particular CPS might be a mediator between obesity and decreased utilisation of CPS and might thus operate as deterring factors [20]. However, we did not assess this and thus did not control for it. Moreover, we neither assessed if participants with obesity perceived the practice equipment as accessible and adequate for people with obesity nor did we assess whether participants with obesity perceived stigmatisation by the practice’s receptionists. Both have been documented before as potential influencing factors [9, 21].

Surprisingly, we found that women with obesity who have a stronger internalised weight bias were found to be more likely to undergo colonoscopies. These results are somewhat counterintuitive and must be interpreted with caution. The apparent contradiction is resolved when considering the recommendation to undergo colonoscopy, which is every ten years for women aged 55 and above. We included women aged 20–65, which means that women who have already undergone colonoscopy once were considered in line with German recommendations. Therefore, women who have already undergone colonoscopy and who have perceived the treatment itself as inadequate or HCP’s attitude as negative have not had yet the chance to decide whether to undergo this procedure a second time.

Although data concerning CPS behaviour might be biased due to self-reporting and social desirability, the external validity should be considered sufficient because of the sampling procedure, i.e., a stratified multilevel random sample.

Another finding is that most women with obesity (68.8%) misclassified their weight status as non-obese. In total, 244 women with obesity perceived themselves as only overweight, whereas n = 58 women (11.60%) perceived themselves as slightly overweight and 7 women perceived themselves to be of normal weight. These results match those observed in earlier studies. Freigang et al. reviewed the current literature regarding the misclassification of self-reported weight status [22]. They reported an overall difficulty in accurately categorising weight status among people of different origins and socioeconomic status (SES). In general, they found that underestimation of weight is common among people with overweight and with low SES. A central factor for the tendency to misclassify weight might be the effect of social groups. In Germany, 54% of the adult population is classified as overweight. In addition, 18% of the German adult population have obesity. Overweight and obesity are more pronounced in low SES groups. Considering this background, being overweight or obese might appear “normal” to people in this social environment [22].

## Limitations

Guidelines regarding CPS differ between countries and national healthcare systems [23]. Whereas recommendations for mammography screening are generally consistent, there are differences for cervical CPS regarding the test method, the age of initiation, and screening intervals [23]. For example, CPS guidelines between Germany and the US differ not only regarding starting age and frequency [24–27], but also regarding financial coverage. In Germany, recommended CPS are fully covered by health insurances. In countries in which CPS are not (fully) covered by health care insurance (such as the US), CPS utilisation rates might be different [28] due to stronger SES-based health inequalities. Thus, acknowledging the varying recommendations for CPS and the divergent financial burden for individuals, comparisons regarding the utilisation of CPS between different countries with heterogeneous healthcare systems must be made carefully.

Further, women who have an increased genetic risk for cancer are recommended and offered CPS more frequently. Unfortunately, we did not capture data on whether participants are at high cancer risk based on genetic factors, i.e., if cases of cancer appear in the family history. Investigating the utilisation of CPS in this particularly vulnerable group is an important issue for future research. Instead, we did ask participants whether they know someone in their”close environment” who has been diagnosed with cancer. Since the question’s wording opens a broad scope for interpretation of how participants define their “close environment” (e.g., family, friends, neighbourhood, colleagues, etc.), this cannot be considered a sufficient proxy variable for participants’ genetic risk. This question was rather asked to assess participants’ awareness of cancer and thus their awareness of the importance of cancer prevention screening.“

Unfortunately, the study did not include some potential influencing factors, such as emotions and thoughts that are associated with CPS. Some forms of CPS can be classified as rather intimate examinations. It is therefore possible that feelings such as shame, fear, and stress are associated with these CPS. Moreover, such negative feelings might also be an expression of (internalized or perceived) weight bias.

This study is also limited by not assessing data regarding the utilisation of contraceptives. Certain forms of contraceptives (e.g., the birth control pill) are only available on prescription, which needs to be renewed every 3 to 6 months. This, in turn, might reinforce the interaction between patients and their gynaecologists and thus the utilisation of CPS. Women who are not using such contraceptives, on the other hand, are not necessarily in constant contact with their gynaecologists. Future studies should therefore consider this factor as a potential confounding variable. Moreover, people with a BMI higher than 40 kg/m^2^ (i.e., morbid obesity) might be less mobile, which could make it difficult for them to get to healthcare practices. However, we did neither assess whether participants were restricted mobility-wise nor did we assess the practices' accessibility and their proximity to public transportation. Unfortunately, the study did not assess how many pregnancies the participants have had. Pregnancies are likely to influence someone’s body consciousness and might therefore be a relevant influencing factor.

We did also not assess participants’ sexual orientation, a potentially relevant factor for CPS utilization. Although for Germany such data is lacking, previous international studies have found that non-heterosexual women were less likely to undergo CPS due to e.g., the fear of getting stigmatized because of their sexual preferences, a lack of information or misperceptions regarding gynaecological CPS [29–31].

Our results indicate that married participants were more likely to undergo CPS. It can be assumed that the emotional support of partners has a positive impact on one’s health care utilisation. That being said, enquiring about partnership status, and not only marital status might have brought additional insight. Further studies should take these variables into account.

It might also be discussed that the data assessed are based on participants’ self-reports and might therefore be biased or inaccurate with special regard to self-reported body weight and body height. Although data might be biased due to self-reporting, we consider the subjectively perceived situation primarily toward experienced weight-based discrimination and internalisation of weight bias as particularly important.

Data on the utilisation of colonoscopies are rather difficult to classify. The results of the Chi-square test and logistic regression analysis indicate (bordering statistical significance) that women with obesity are less likely to undergo colonoscopies. However, since we assessed data of women aged 20–65 years and colonoscopies are offered and recommended every ten years for women aged 55 and above, data on this particular CPS behaviour is rather difficult to interpret. Future studies should consider also recruiting women aged 65 and above to investigate this association in greater detail.

## Conclusion

The purpose of the current study was to provide data concerning the utilisation of gynaecological CPS in women and whether the utilisation differs between women with and without obesity. Moreover, we aimed to assess influencing factors that either promote or suppress CPS behaviour in women with obesity. We found differences in the recommended utilisation of Pap smears and clinical breast examinations between both weight groups, but not in the utilisation of mammography scans, FOBT, and colonoscopies. A sub-analysis with the obese sample revealed that women who have had cancer themselves and knew about CPS forms were more likely to undergo these screenings as recommended. Contrary to our expectations, we did not find perceived obesity-related stigmatisation or discrimination to be significantly associated with CPS behaviour. However, it cannot be completely ruled out that obesity-based stigmatisation might inhibit CPS behaviour. Firstly, we have discussed some potential indications that emphasize weight-based barriers to health care (i.e. self-perceived weight status, WBIS). Secondly, we could find no differences in CPS when it is conducted by women themselves (i.e. self-examination of the breast), which might indicate that women of all weight groups are equally careful. And thirdly, we have discussed methodological shortcomings and limitations regarding the instruments applied or left out that might clarify the association more comprehensively.

Several questions remain to be answered. Considerably more work will thus need to be done to determine why women with obesity have a higher risk of avoiding cervical and some kind of breast CPS. Also, future studies need to be carried out to evaluate the previously established changes in cancer prevention programs and if and how these reformations affect the CPS behaviour of women with obesity.

## Supplementary Information

**Additional file 1:** Questionnaire (translated version).

## Data Availability

The datasets used and/or analysed during the current study are available from the corresponding author upon reasonable request.

## Abbreviations

BMI: Body mass index
CPS: Cancer prevention screenings
FOBT: Faecal occult blood test
HCP: Health care professionals
HPV: Human papillomavirus
Pap: Papanicolaou
WBI: Weight bias internalisation
