# Supplementary material for: Cancer prevention in females with and without obesity: Does perceived and internalised weight bias determine cancer prevention behaviour?
Source: BMC Womens Health. 2022 Dec 9;22:511. doi: 10.1186/s12905-022-02085-2 (PMC9733287; doi:10.1186/s12905-022-02085-2)
Supplement: Supplementary file 1 — Additional file 1: Questionnaire (translated version). [file 12905_2022_2085_MOESM1_ESM.docx]

# Questionnaire

## Cancer prevention in females with and without obesity –

## Does perceived and internalised weight bias determine cancer prevention behaviour?

| Sample | Questions | Answer categories |
| --- | --- | --- |
| Cancer Screening Behavior | | |
| If age ≥ 20 | - For women aged 20 and above a Pap smear test is classified as a cancer prevention screening offered and financed by health insurances. How often do you use the Pap smear test? | - Never - less than once a year - once a year - twice a year - more than twice a year - Prefer not to say |
| If age ≤ 30 | - Have you had an HPV vaccination? | - Yes - No - Prefer not to say |
| If age ≥30 | - For women aged 30 and above a clinical breast examination conducted by a gynecologist is classified as a cancer prevention screening, which is offered and financed by health insurances. How often do you use the clinical breast examination? | - Never - less than once a year - once a year - twice a year - more than twice a year - Prefer not to say |
| Full sample | - Do you examine your breasts yourself? | - Yes - No - Prefer not to say |
| Full sample | - If yes, how often do you self-examine your breast? | - daily - several times a week - once a week - several times a month - once a month - less than once a month - infrequent - I do not know - I prefer not to say |
| If age ≥50 | - For women aged 50 and above a mammography screening is classified as a cancer prevention screening, which is thus offered and financed by health insurances. Have you ever used a mammography screening? | - Yes - No - Prefer not to say |
| If age ≥50 | - If yes, how often did you use a mammography screening? | - Once - Twice - Three times - Four times - Five times - More than five times - I do not know - I prefer not to say |
| If age ≥50 | - For women aged 50 and above a fecal occult blood test is classified as a cancer prevention screening, which is thus offered and financed by health insurances. Have you ever used a fecal occult blood test? | - Yes - No - Prefer not to say |
| If age ≥50 | - If yes, how often did you use a fecal occult blood test? | - Once - Twice - Three times - Four times - Five times - More than five times - I do not know - I prefer not to say |
| If age ≥50 | - Have you ever used a colonoscopy? | - Yes - No - Prefer not to say |
| If age ≥50 | - If yes, how often did you use a colonoscopy? | - Once - Twice - Three times - Four times - Five times - More than five times - I do not know - I prefer not to say |
| Weight Status | | |
| Full sample | - Please state your body height and current body weight | - Open answer category |
| Full sample | - How do you perceive yourself? Would you consider yourself to be underweight, of normal weight, overweight, or obese? | - Extremely underweight - underweight - Slightly underweight - Normal weight - Slightly overweight - overweight - Extremely overweight - I do not know - I prefer not to say |
| Experienced Weight Bias | | |
| Full sample | - The following questions are about your experiences with health care professionals of different disciplines. Have you ever felt inadequately treated by the following doctors? - Physicians - Gynecologists - Proctologists - Dermatologists - Dentists - Orthopedists | - Yes - No - I do not know - Prefer not to say |
| If bmi > 30 | - Have you ever felt excluded or discriminated because of your body weight? | - Yes - No - I do not know - Prefer not to say |
| If bmi > 30 | - Have you ever felt excluded or discriminated because of your body weight by a health care professional of the following disciplines? - Physicians - Gynecologists - Proctologists - Dermatologists - Dentists - Orthopedists | - Yes - No - I have not visited yet - I do not know - Prefer not to say |
| Internalized Weight Bias | | |
| If bmi > 30 | - In the following you hear some statements how people might feel about their weight. Please state to what extent you agree with the statement on a scale from 1 (I completely disagree) to 7 (I completely agree). - As an overweight person, I feel that I am just as competent as anyone. - I am less attractive than most other people because of my weight. - I feel anxious about being overweight because of what people might think of me. - I feel anxious about being overweight because of what people might think of me. - Whenever I think a lot about being overweight, I feel depressed. - I hate myself for being overweight. - My weight is a major way that I judge my value as a person. - I don’t feel that I deserve to have a really fulfilling social life, as long as I’m overweight. - I am OK being the weight that I am. - Because I’m overweight, I don’t feel like my true self. - Because of my weight, I don’t understand how anyone attractive would want to date me. | - strongly disagree - disagree - slightly disagree - neither agree nor disagree - slightly agree - agree - strongly agree - I do not know - I prefer not to say |
| Cancer awareness | | |
| If age ≥ 20 | - Health insurances provide cancer prevention screenings. Which gynecological cancer prevention screenings that are conducted by gynecologists are offered and financed for women aged 20 and above? | - [Do not read loud; multiple answers possible] - Pap smear test - ultrasound examination - Clinical examination of the breast - mammography screenings - others: note - Prefer not to say |
| If age ≤ 30 | - Did you know that there is a vaccination for Human Papilloma Viruses (HPV) that protects against cervical cancer? | - Yes, I knew that - No, I did not know that - Prefer not to say |
| If age ≥30 | - From the age of 30, health insurances provide and finance another cancer prevention screening. Do you know which cancer prevention screening that is? | [Do not read loud; multiple answers possible]   - Pap smear test - ultrasound examination - Clinical examination of the breast - mammography screenings - others: note - Prefer not to say |
| If age ≥50 | - From the age of 50, health insurances provide and finance another cancer prevention screening. Do you know which cancer prevention screening that is? | [Do not read loud; multiple answers possible]   - Pap smear test - ultrasound examination - Clinical examination of the breast - mammographie screenings - others: note - Prefer not to say |
| If age ≥50 | - From the age of 50, health insurances provide and finance colorectal cancer prevention screening. Do you know which additional colorectal cancer prevention screening that are offered? | [Do not read loud; multiple answers possible]   - Colonoscopy - Fecal occult blood test (FOBT) - Others: note - Prefer not to say |
| Full sample | - Have you been diagnosed with a cancer disease that is currently gone? | - Yes - No - Prefer not to say |
| Full sample | - Are you currently been diagnosed with any caner disease? | - Yes - No - Prefer not to say |
| Full sample | - Has someone in your social environment (e.g., family members, friends, colleagues) ever been diagnosed with a cancer disease? | - Yes - No - Prefer not to say |
| Full sample | - Have you ever had a benign tumor disease that is currently gone? | - Yes - No - Prefer not to say |
| Confounding variables | | |
| Full sample | - Are you statutorily or privately health insured? | - Statutorily health insured - Privately health insured - Prefer not to say |
| Full sample | - In which year are you born? | - Prefer not to say |
| Full sample | - What is your highest educational degree? | - Still in school - No degree - Secondary school I - Secondary school II - Advanced technical college certificate - Higher education (university degree) - Other - Prefer not to say |
| Full sample | - Are you currently employed? | - Yes - No - Prefer not to say |
| Full sample | - Please state your marital status | - Single - Married - Married but separated - Divorced - Widowed - Prefer not to say |
| Full sample | - How many people live in your household? | - Prefer not to say |
| Full sample | - How much is your monthly household income? | - Less than 500 Euros a month - 500 < 1000 Euros a month - 1000 < 1500 Euros a month - 1500 < 2000 Euros a month - 2000 < 2500 Euros a month - 2500 < 3000 Euros a month - 3000 < 3500 Euros a month - 3500 < 4000 Euros a month - 4000 < 4500 Euros a month - ≥ 4500 Euros a month - I do not know - Prefer not to say |
| Full sample | - What is your marital status? | - Married and living together - Married and living separately - Single - Divorced - widowed |
